# Elimination of Unwanted Modes in Wavelength-Selective Uncooled Infrared Sensors with Plasmonic Metamaterial Absorbers using a Subtraction Operation

**DOI:** 10.3390/ma12193157

**Published:** 2019-09-27

**Authors:** Shinpei Ogawa, Yousuke Takagawa, Masafumi Kimata

**Affiliations:** 1 Advanced Technology R&D Center, Mitsubishi Electric Corporation, 8-1-1 Tsukaguchi-Honmachi, Amagasaki, Hyogo 661-8661, Japan; 2 College of Science and Engineering, Ritsumeikan University, 1-1-1 Noji-higashi, Kusatsu, Shiga 525-8577, Japan; Takagawa.Yosuke@ds.MitsubishiElectric.co.jp (Y.T.); kimata@se.ritsumei.ac.jp (M.K.)

**Keywords:** plasmonics, metamaterials, uncooled, IR sensors, wavelength-selective

## Abstract

Wavelength- or polarization-selective uncooled infrared (IR) sensors have various applications, such as in fire detection, gas analysis, hazardous material recognition, biological analysis, and polarimetric imaging. The unwanted modes originating due to the absorption by the materials used in these sensors, other than plasmonic metamaterial absorbers (PMAs), cause serious issues by degenerating the wavelength or polarization selectivity. In this study, we demonstrate a method for eliminating these unwanted modes in wavelength- or polarization-selective uncooled IR sensors with various PMAs, using a subtraction operation and a reference pixel. The aforementioned sensors and the reference pixels were fabricated using a complementary metal oxide semiconductor and micromachining techniques. We fabricated the reference pixel with the same structure as the PMA sensors, except a flat mirror was formed on the absorber surface instead of PMAs. The spectral responsivity measurements demonstrated that single-mode detection can be achieved through the subtraction operation with the reference pixel. The method demonstrated in this study can be applied to any type of uncooled IR sensors to create high-performance wavelength- or polarization-selective absorbers capable of multispectral or polarimetric detection.

## 1. Introduction

Uncooled infrared (IR) sensors with micro-electro-mechanical system (MEMS) based pixel structures are used in a wide range of applications, such as in security, surveillance, maintenance, firefighting, and in the automotive industry [1,2]. Recently, there has been increased interest in developing advanced functional uncooled IR sensors with wavelength- or polarization-selectivity, especially due to their applicability in fire detection, gas analysis, hazardous material recognition, biological analysis, and polarimetric imaging [3,4]. The wavelength-selective function is used for analyzing and recognizing objects by spectral information, and the polarization-selective function can contribute to enhancing object recognition, such as face recognition using polarimetric information [5]. We have previously demonstrated wavelength- or polarization-selective uncooled IR sensors using various plasmonic metamaterial absorbers (PMAs), such as plasmonic crystal (PC) [6,7,8,9], metal-insulator-metal (MIM) [3,10], and mushroom-type PMAs [11,12,13] at the middle-wavelength IR (MWIR) and the long-wavelength IR (LWIR) regions. The PMAs have periodic metal surface structures, which support the induced surface plasmon modes and lead to the wavelength- or polarization-selective absorption. Therefore, the absorption wavelength and the polarization can be structurally controlled by the surface patterns of these structures. Such PMAs can realize wavelength- or polarization-selective functions in uncooled IR sensors without filters or polarizers, which lead to low-cost fabrication and enable different pixels to be integrated in an array. Moreover, PMAs with smaller and thinner absorbers have significant advantages over traditional absorbers.

Several researchers have studied the effects of various surface patterns of PMAs to realize single [14,15,16,17], multi-mode [18,19,20,21,22,23], and broadband absorption [24,25,26,27,28] in broad-wavelength regions ranging from ultraviolet [29] to terahertz [30,31]. However, only a few investigations have been performed that demonstrate the application of PMAs in actual devices such as uncooled IR sensors [32]. In some cases, unwanted absorption modes have been observed, which cause additional absorption of wavelengths, and therefore degenerated performance with regard to wavelength or polarization selectivity. These unwanted modes cannot be attributed to the propagating or localized surface plasmon resonance induced by the surface patterns of the PMAs. It is extremely important to eliminate such unwanted absorption modes to realize high-performance wavelength- or polarization-selective uncooled IR sensors. In this study, we investigate the origin of unwanted absorption modes and develop a method to eliminate such absorption modes.

## 2. Elimination of Unwanted Modes 

### 2.1. Origin of Unwanted Modes

**Figure 1:**
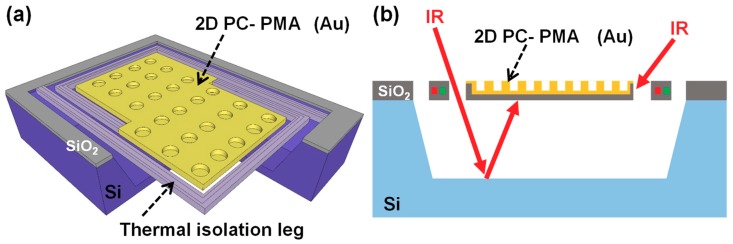
Schematic illustrations of (**a**) typical micro-electro-mechanical system (MEMS) based thermopile with 2D PC-PMA and (**b**) unwanted absorption at the backside and the sides of the 2D PC-PMA.

Figure 1a and b shows schematic illustrations of a typical thermopile sensor with the cross-section of PMAs as uncooled IR sensors and the side view of the PMA sensor with unwanted IR absorptions, respectively. In this study, we adopted two-dimensional (2D) PC-type PMAs (2D PC-PMAs) with a thermopile [33] as a MEMS-based uncooled IR sensor. A 2D PC-PMA produces wavelength-selective absorption at a wavelength nearly equal to its surface period [6,7].

We had previously demonstrated that the unwanted absorption occurs owing to the absorptions at the backside and other sides of SiO_2_ in a 2D PC-PMA (Figure 1b). To address this issue, an Al reflection layer was inserted at the backside and the sides of the 2D PC-PMAs, which were coated with Au. This drastically reduced unwanted absorption in the system [7]. Additionally, clear wavelength or polarization selectivity was achieved by restricting the incident IR ray to only the absorber area using a pinhole. Without the pinhole, we could still observe unwanted absorption in the LWIR region. 

**Figure 2:**
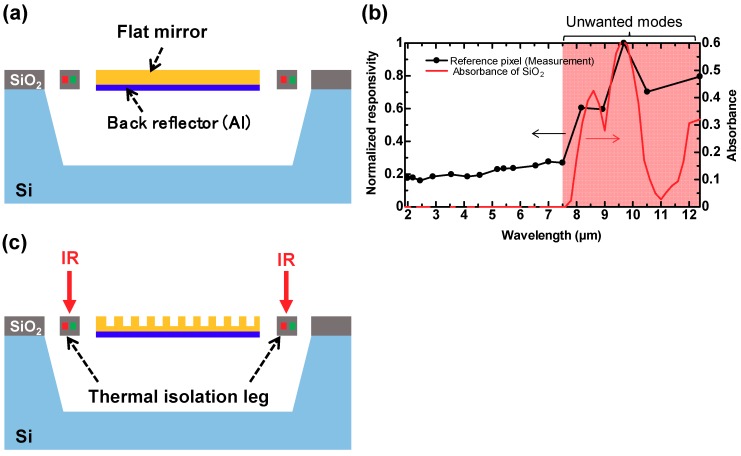
(**a**) Schematic illustration of a typical MEMS-based thermopile with flat mirror (reference pixel) and (**b**) its measured spectral responsivity and calculated absorbance of 1.5 μm thick SiO_2_. (**c**) Schematic illustration of unwanted absorption by the SiO_2_-thermal isolation legs in typical MEMS-based uncooled IR sensors.

A schematic of the thermopile with the flat mirror surface (reference pixel) prepared in this study is shown in Figure 2a. Its responsivity and absorbance were measured to investigate the origin of these unwanted modes. Figure 2b shows the measured spectral responsivity of the reference pixel and the calculated value of absorption by the 1.5 µm thick SiO_2_, which corresponds to the actual thickness of the sensors, as shown in Figure 2a. The details of the fabrication procedure and the measurement systems are explained in subsequent sections. As expected, the reference pixel produced no output signal because the surface mirror and the backside reflector reflected all the incident IR rays, and there was no absorption of IR rays. The calculations were performed using the rigorous coupled wave analysis (RCWA) method [34]. RSOFT DiffractMOD software (version 2018.12-1, Synopsys, Inc., California, CA, USA) was used for the RCWA calculation. Unexpectedly, responsivity peaks were observed in the LWIR region, which corresponded to the calculated absorbance of SiO_2_. The calculated absorbance spectra were attributed to the thin-film interference in the thickness direction of SiO_2_. These results demonstrate that the origin of the unwanted modes can be attributed to the intrinsic absorption of SiO_2_ used in the sensor structures as the thermal isolation legs (Figure 2c). 

The thermal isolation legs are typically used in most MEMS-based uncooled IR sensor structures. Therefore, the unwanted absorption by the thermal isolation legs is a common obstacle to realizing high performance in wavelength- or polarization-selective uncooled IR sensors. It is extremely difficult to block the top surface, the sides, and the backside of the thermal isolation legs to avoid any unwanted absorption in these sensors, and therefore we present a subtraction operation for eliminating the unwanted modes.

### 2.2. Subtraction Operation Using Reference Pixel

**Figure 3:**
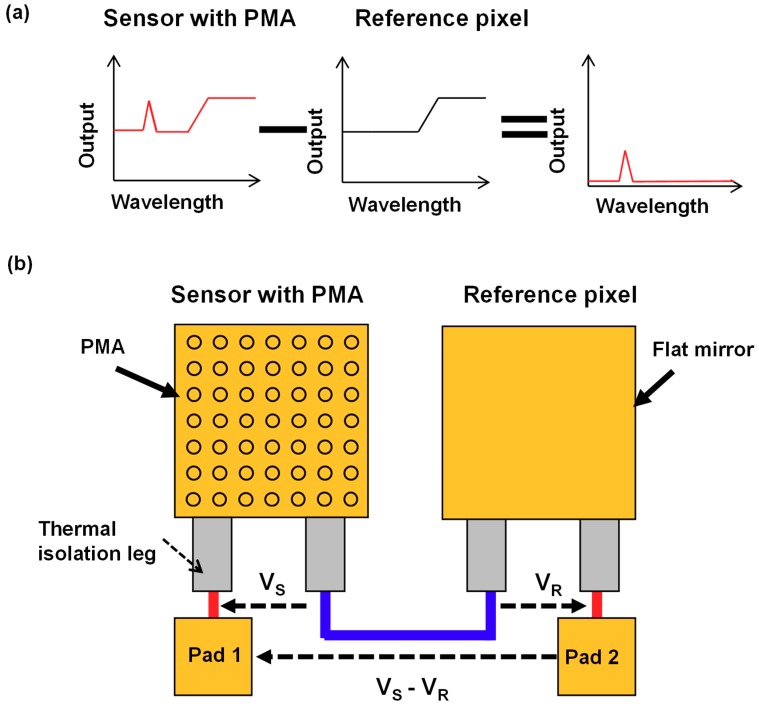
(**a**) Demonstration of the concept to eliminate the unwanted modes using a subtraction operation with a reference pixel. (**b**) Configuration for the subtraction operation between a thermopile with PMA and a reference pixel with flat mirror.

Figure 3a shows the proposed concept to eliminate unwanted modes using a subtraction operation with the reference pixel. The sensor with PMAs has a wavelength-selective detection peak caused by the plasmonic resonance in PMAs and relatively broad detection in the LWIR region owing to the absorption by the SiO_2_ present in the thermal isolation legs. The reference pixel was designed with the same sensor structure except for the absorber surface in which a flat mirror was used as the absorber surface instead of PMAs. Therefore, the reference pixel shows the same unwanted absorption modes as the sensor with PMAs, which is caused by the SiO_2_-thermal isolation legs. Figure 3b shows the configuration of the sensor with PMA and the reference pixel with a flat mirror for the subtraction operation. The output signal between Pad 1 and Pad 2 was measured, whereby the subtraction voltage (V_S _–V_R_) between the output voltage of the sensor with the PMA (V_S_) and that of the reference pixel (V_R_) was obtained.

## 3. Sensor Fabrication

**Figure 4:**
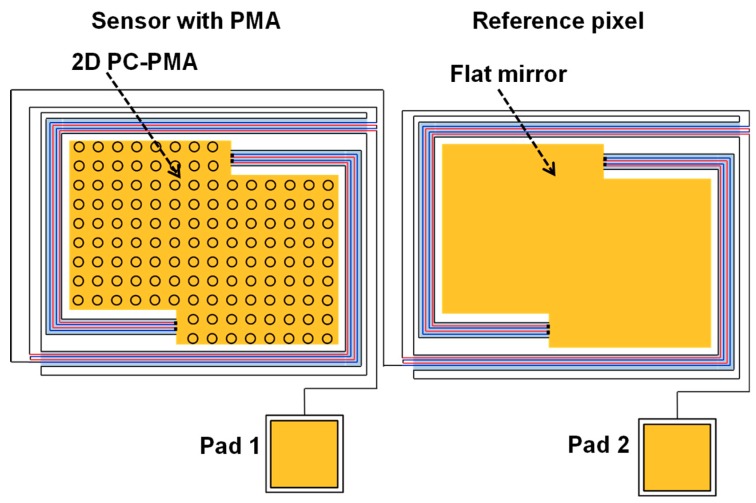
Schematic illustration of the sensor with 2D PC-PMA and the reference pixel, in which the output signal was produced from the subtraction operation between them.

Figure 4 shows the actual configuration of the sensor with 2D PC-PMAs and the reference pixel. The signal lines to measure voltages were connected as shown in Figure 3b.

**Figure 5:**
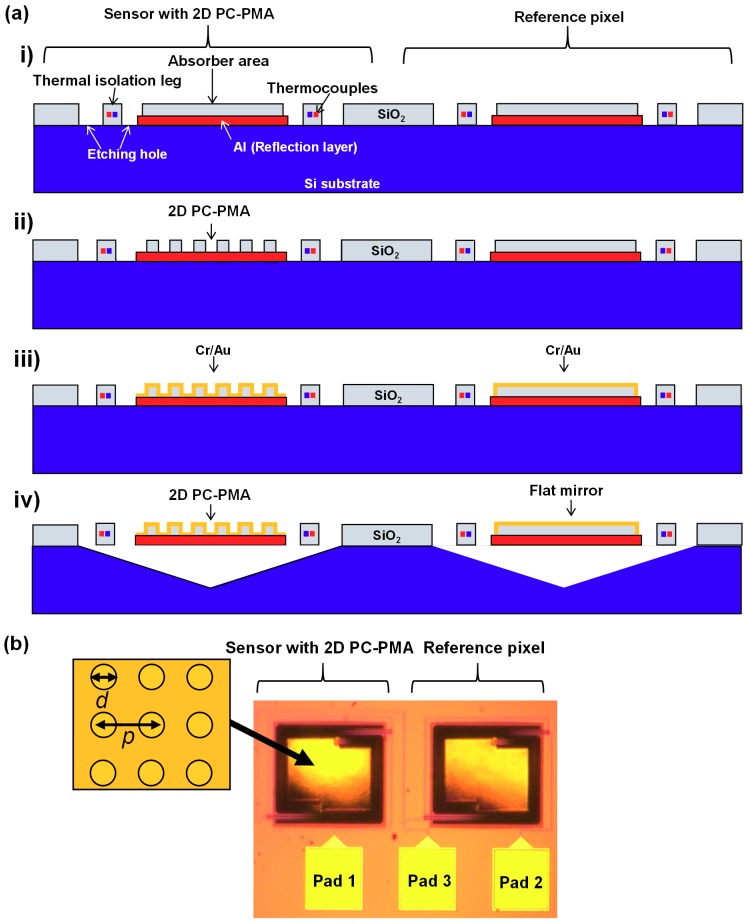
(**a**) Fabrication procedure of a MEMS-based thermopile with PMAs and a reference pixel. (**b**) Optical microscope image of the developed sensor with PMAs and the reference pixel.

Figure 5a shows the fabrication procedure of the sensors with 2D PC-PMAs and the reference pixel with the subtraction operation. The sensors with 2D PC-PMAs and the reference pixels were fabricated on a six inch silicon (Si) substrate using a standard complementary metal oxide semiconductor (CMOS) process, the details of which have been reported in our previous studies [7,8,9,20]. A series of p- and n-type poly-Si regions formed thermocouples, and a selected amount of ion implantation controlled their resistivity. An Al layer was deposited under the absorber area as a backside reflective layer, and holes for cavities were formed via reactive-ion etching (RIE). Subsequently, a 1.5 µm thick SiO_2_ layer was deposited on the absorber area (Figure 5a, i). Following this, the 2D PC-PMA structures were fabricated over the SiO_2_ layer of the IR absorber area of the sensors by using the RIE process (Figure 5a, ii), whereas no pattern was formed in the reference pixels. Next, a 50 nm thick Cr adhesive layer and 250 nm thick Au surface plasmon resonance layer were deposited by sputtering on the entire surface of the wafer. The Au layer was sufficiently thicker than the skin depth of the IR wavelengths, therefore, absorption of the incident IR rays by the Cr and SiO_2_ layers beneath the Au layer could not occur. The Cr and Au layers were selectively etched using a wet etchant, except the part of the layers covering the 2D PC-PMA and flat mirror regions. (Figure 5a, iii). We confirmed that the Cr and Au layers were uniformly coated using scanning electron microscopy observation. Each wafer was then diced into chips. The Si of every chip was anisotropically etched through the holes using tetramethylammonium hydroxide (TMAH), as shown in Figure 5a, iv. The backside reflective Al layer was not etched because TMAH was doped with Si. The thermally isolated freestanding structure was completed in the cavity under the IR absorber area.

Figure 5b shows the optical image of the fabricated sensor with 2D PC-PMA and reference pixel. The absorber area is 300 × 200 μm^2^. Pad 3 (in Figure 5b) was added in the actual system for measuring the output voltage of the sensor with the PMAs (V_S_) and the reference pixel (V_R_) independently, as shown in Figure 3b. It should be noted that the surface pattern of the 2D PC-PMA is too fine to be observed using an optical microscope. The diameter and the period of the dimples in 2D PC-PMA were defined as d and p, respectively. The depth of the dimples was fixed at 1.5 μm in this study.

We fabricated three kinds of the 2D PC-PMA sensors with d and p values of 4.0 and 5.0 μm, 4.0 and 5.5 μm, 4.0 and 6.0 μm, 4.0 and 6.5 μm, and 7.0 and 8.0 μm, which are labeled as sensors A, B, C, D, and E, respectively. The same reference pixel was used for comparison with each sensor. The sensors, A, B, C, D, and E, were expected to have wavelength-selective absorptions at 5.0, 5.5, 6.0, 6.5, and 8.0 μm, respectively, depending on their surface period.

## 4. Measurement and Results

To measure the spectral responsivity, the sensors with 2D PC-PMAs and the reference pixel were set in a vacuum chamber with a Ge window. The sensors with 2D PC-PMAs and the reference pixel were irradiated simultaneously with IR radiation from a blackbody at a temperature of 1000 K through a narrow bandpass filter. The incidence angle was normal to the 2D PC-PMA sensor and the reference pixel. The output voltages such as V_S_, V_R_, and (V_S _–V_R_) were monitored using a computer. The responsivity (V/W) was calculated as the ratio between the difference in the output voltage for the on and off states and the input power. The measurement system used in this study is the same as the one reported in our previous studies [6,7,8,9,20], except for the use of pinholes included in this study. The input power was calculated from the spectral radiant emittance equation at the evaluated wavelength, with the measurement system parameters such as transmittance of the IR ray power from the blackbody to the sensor through the air, narrow bandpass filters and the Ge window, and the absorber area of the sensors, as previously reported [6,7,8,9,20].

**Figure 6:**
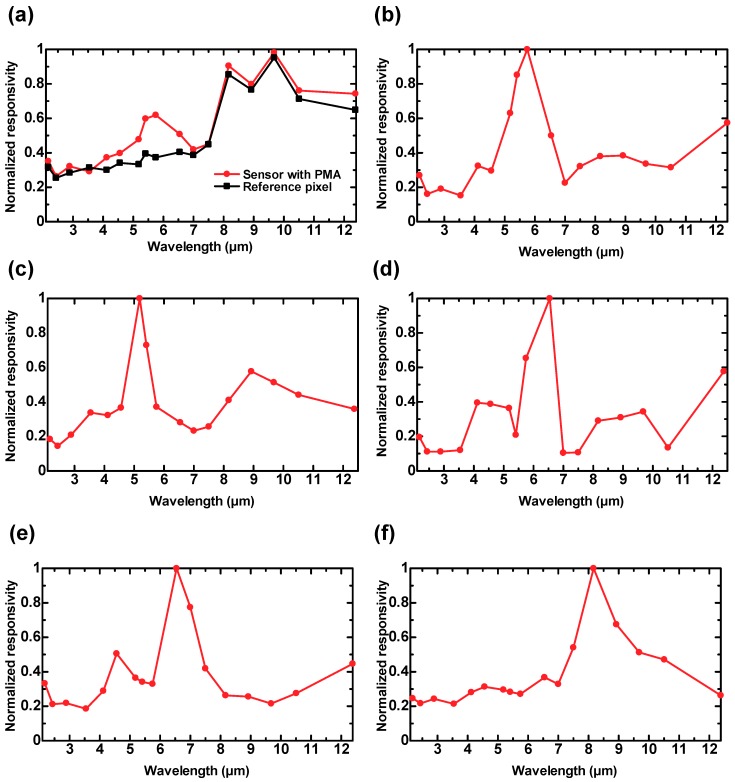
Normalized spectral responsivity: (**a**) of the sensor with PMA and the reference pixel, (**b**) after the subtraction operation for sensor B (*p* = 5.5 μm and d = 4.0 μm), (**c**) after the subtraction operation for sensor A (*p* = 5.0 μm and d = 4.0 μm), (**d**) after the subtraction operation for sensor C (*p* = 6.0 μm and d = 4.0 μm), (**e**) after the subtraction operation for sensor D (*p* = 6.5 μm and d = 4.0 μm), and (**f**) after the subtraction operation for sensor E (*p* = 8.0 μm and d = 7.0 μm).

Figure 6a,b shows the normalized spectral responsivity of sensor B and the reference pixel, and the normalized spectral responsivity after the subtraction operation, respectively. It is worth noting that the responsivity was normalized with respect to the maximum value in order to avoid any misleading results because the absorption peak caused by surface plasmon resonance was so sharp that wavelength resolution of the measurement system was not sufficient to precisely determine the maximum value. Nevertheless, we confirmed that the developed sensor exhibited comparable responsivity to conventional thermopiles. For example, a maximum responsivity of 100 V/W was achieved. Figure 6a shows clearly that the unwanted modes were produced in the LWIR region, and the wavelength-selective detection was degenerated by these unwanted modes. The wavelength-selective detection was clearly observed at 5.5 μm after the subtraction operation, as evident in Figure 6b. Figure 6c–e show the normalized spectral responsivity of sensors A, C, D, and E with the subtraction operation, and the wavelength-selective detection was clearly observed at approximately 5.0 μm, 6.0 μm, 6.5 μm, and 8.0 μm, respectively. It should be noted that the normalized responsivity peak of sensor C can be considered to be at 6.0 μm because the peak wavelength can be determined at between 5.5 μm and 6.5 μm due to the asymmetric shape of the peak. The five detected peak wavelengths for each sensor are approximately equal to the *p*-values, which corroborates the theoretical results [6,7]. These results present direct evidence that the unwanted modes were successfully eliminated using the subtraction operation with the reference pixel.

## 5. Conclusions

In this study, we present a method for the elimination of unwanted modes in a wavelength-selective uncooled IR sensor, using a subtraction operation with a reference pixel. To this end, MEMS-based wavelength-selective uncooled IR sensors using 2D PC-PMAs with reference pixels were fabricated using CMOS and micromachining techniques. The reference pixel possessed the same sensor structure, except a flat mirror was used on the absorber surface instead of PMAs. The spectral responsivity measurements demonstrated that single-mode detection was achieved through the subtraction operation with a reference pixel. It is important to consider the absorptions in other regimes by the PC-PMA-based uncooled IR sensors from an application standpoint. The concept demonstrated in this study can be applied to any type of uncooled IR sensor, such as bolometers [35], silicon-on-insulator diodes [36,37], ferroelectrics [38], and photomechanical sensors [39,40] with wavelength- or polarization-selective absorbers that are capable of multispectral or polarimetric detection, and also to any type of PMAs such as PC, MIM, mushroom, and other types of structures.
